# Multifunctional movable-type coding metasurface enabling reconfigurable diffractive neural networks

**DOI:** 10.1038/s41377-026-02216-6

**Published:** 2026-02-26

**Authors:** Zhicai Yu, Xinyu Li, Ze Gu, Long Chen, Jianlin Su, Zixuan Cai, Xinyi Yu, Shilong Qin, Lei Zhang, Qian Ma, Jian Wei You, Tie Jun Cui

**Affiliations:** 1 State Key Laboratory of Millimeter Wave, Southeast University, Nanjing, China; 2 School of Information Science and Engineering, Southeast University, Nanjing, China

**Keywords:** Metamaterials, Displays, Microwave photonics

## Abstract

Optical computing holds significant promise across diverse applications due to its low latency, power efficiency, and multidimensional processing capabilities. However, current diffraction neural networks (DNNs) generally lack reconfigurability, limiting the scalability of the optical computing systems. Inspired by movable-type printing technology, here we propose a movable-type coding metasurface to enable multiple functionalities such as electromagnetic (EM) computing, holography, and sensing. By cascading multiple layers of the proposed metasurfaces, we further develop a movable-type reconfigurable DNN (MT-RDNN). It can be seamlessly adapted from handwritten digit to letter classification tasks by replacing the meta-atoms in the last hidden metasurface layer. Moreover, a single-layer movable-type coding metasurface can be reconfigured to perform EM holography and multi-person vital sign sensing through modular meta-atom rearrangement. Featuring simple reconfiguration, high flexibility, and modular scalability, the proposed movable-type coding metasurface enables versatile and reusable EM computing, holography, and sensing applications.

## Abstract (web summary)

Inspired by movable-type printing, we propose movable-type coding metasurfaces for reconfigurable DNN in computing, holography, and sensing.

## Graphical abstract

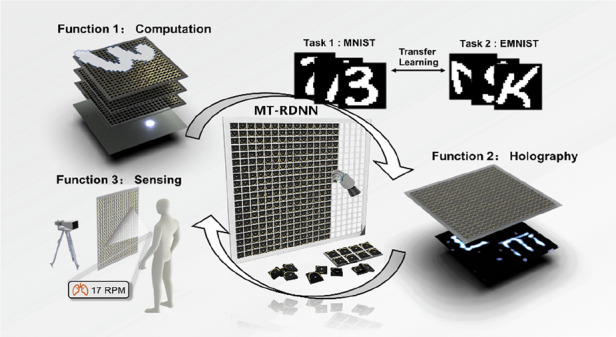

## Introduction

As artificial electromagnetic (EM) media^1^, metamaterials witnessed remarkable advancements over the past two decades owing to their exceptional capability to manipulate EM waves, including phase^2,3^, amplitude^4,5^, wavelength^6–8^, and polarization^9–12^. In particular, the advent of programmable or reconfigurable metamaterials and metasurfaces^13–16^ has opened new avenues for information processing^17^ and intelligent systems^18^, enabling a broad range of applications such as wireless communication^19^, smart sensing^20,21^, adaptive control^22,23^, and neuromorphic computing^24,25^. Among these advances, diffractive neural networks (DNNs)^26–30^ operating in the microwave regime emerged as a promising frontier for intelligent EM computing. Inspired by the optical counterparts^31,32^, microwave DNNs have rapidly evolved across various platforms, including spatial architectures, planar structures^33^, and spoof surface plasmon polariton (SSPP)^34,35^ configurations. These systems enable a wide range of functions, including logic operations^36,37^, object detection^38^, dynamic tracking^39^, and angle-of-arrival estimation^40^, highlighting the significant potential of microwave-based neural networks in next-generation intelligent EM systems.

The current research on metasurface for EM computing is primarily focused on photoelectric^41–43^ and thermal modulation techniques^44,45^, which alter the optical response of meta-atoms embedded in stimuli-responsive materials through electrical, thermal, or optical stimulation^46^. However, these approaches are constrained by tailoring materials for specific frequency bands. Meanwhile, multifunctional reconfigurable metasurfaces often suffer from high costs, complex fabrication processes, high power consumption, and intricate control systems, hindering their applications in EM diffractive neural networks. In contrast, the mechanically reconfigurable metasurfaces^47–49^ provide a simpler and more versatile solution to dynamic EM control, achieving reconfigurability through basic mechanical operations such as meta-atom rotation^41^ or flipping^42^. Despite the advantages of mechanical control, prior research has predominantly focused on manipulating surface and reflected waves using mechanically reconfigurable metasurfaces. Transmissive and cascaded mechanical metasurfaces have received comparatively limited attention, although they are essential for EM computing. Moreover, the existing designs of mechanically reconfigurable metasurfaces impose strict requirements on the rotation angles of meta-atoms, complicating the reconfiguration process and hampering their rapid deployment. Therefore, it is essential to develop mechanically reconfigurable metasurfaces that enable flexible assembly and reuse of meta-atoms, thereby enhancing both efficiency and scalability of DNNs.

Transfer learning^50^ is a fundamental concept in machine learning, where knowledge acquired from a source task is transferred and applied to a new task, thereby reducing dependence on large datasets and prolonged training. In recent years, this concept has been gradually extended to the physical layer^51^, where partial replacement or adjustment of hardware structures can be employed to avoid retraining from scratch. However, most existing work^32,47,52^ was achieved by replacing certain layers within the DNN. Although layer-level replacement strategies can reduce the time required for functional switching, they inherently restrict the reconfigurable granularity of DNNs, leading to substantial fabrication costs. Additionally, they provide limited reusability and scalability, since the number of meta-atoms per layer is fixed during fabrication and cannot be flexibly adjusted to meet varying task requirements. Therefore, extending transfer learning to the unit-level, where meta-atoms can be independently reconfigured, is particularly desirable. This paradigm not only improves the efficiency of functional switching but also enhances structural flexibility and scalability, establishing a foundation for efficient, low-cost, and reconfigurable DNNs.

Movable-type printing is a revolutionary printing technique originating in ancient China, which used characters cast in metal or carved from wood as reusable molds that could be arranged and reused to compose different pages of text. Inspired by the modularity and reusability of this technology, we propose a reconfigurable transmissive metasurface made of detachable meta-atoms. Eight categories of meta-atoms with distinct EM responses are designed to serve as the functional “molds” of the metasurface. These meta-atoms can be mechanically reassembled into customized configurations, thus significantly enhancing the efficiency of metasurfaces and reducing the production costs. Leveraging its reconfigurable and reusable characteristics, the proposed movable-type coding metasurface enables multiple EM functions, particularly to construct a movable-type reconfigurable diffractive neural network (MT-RDNN) by cascading multiple metasurface layers. MT-RDNN can be rapidly adapted to new tasks by replacing only a few meta-atoms, showing excellent task flexibility and reconfigurability. Furthermore, the single-layer movable-type coding metasurface also enables flexible wavefront shaping and supports various EM tasks, such as holography and contactless human vital-sign sensing. For instance, by selectively replacing a subset of meta- atoms, the EM wave can be dynamically focused on different spatial locations to enhance the signal-to-noise ratio (SNR) for vital sign detection. This modular and reconfigurable design paradigm improves the versatility, reusability, and user-friendliness of metasurfaces and microwave DNNs significantly, offering a promising route toward low-power and task- adaptive intelligent EM systems.

## Results

**Fig. 1:**
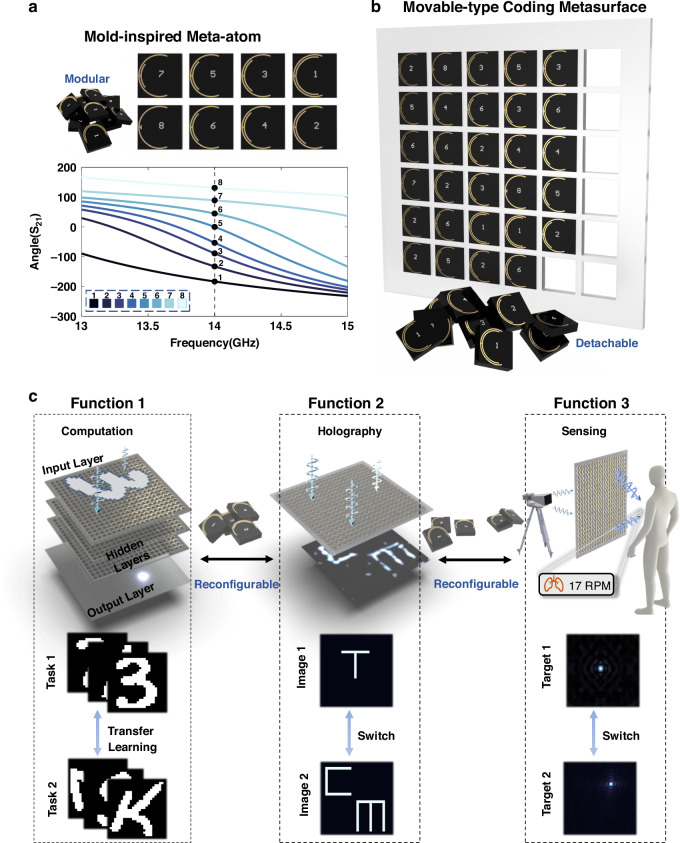
Movable-type reconfigurable metasurface for multiple EM functions. **a** Mold-inspired meta-atoms from eight categories. **b** Schematic illustration of the proposed 3-bit movable-type coding metasurface. **c** Multiple functions enabled by the proposed movable-type coding metasurface, including an MT-RDNN for EM computing, EM holography, and contactless human vital sign sensing. MT-RDNN consists of one input layer, three movable-type coding metasurface layers, and one output layer

As shown in Fig. 1, the proposed movable-type coding metasurface integrates the modularity of movable-type printing with programmability of metasurfaces. We design eight categories of mold-inspired transmissive meta-atoms working at 14 GHz, each of which has unique EM response, as presented in Fig. 1a. Then, the eight categories of meta-atoms can be assembled in arbitrary configurations, forming a 3-bit movable-type coding metasurface (Fig. 1b). Due to its reconfigurable nature, the proposed metasurface can achieve a variety of EM functions, including holography, human vital sign sensing, and construction of an MT-RDNN through multilayer cascading, as shown in Fig. 1c.

### Design and fabrication of mold-inspired meta-atom

**Fig. 2:**
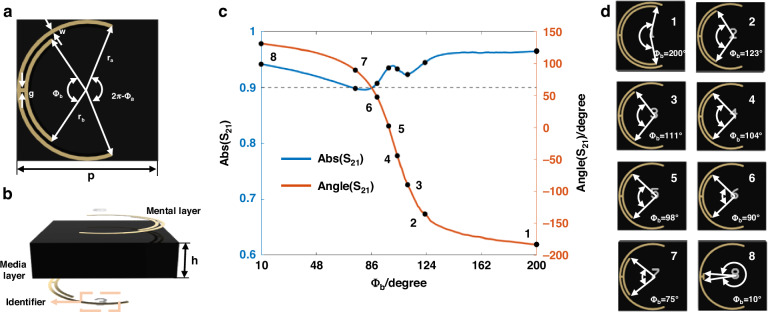
Mold-inspired meta-atom. **a** Top view of the designed meta-atom. The structural parameters are: p = 12 mm, r_a_ = 5.8 mm, r_b_ = 5.2 mm, w = 0.3 mm, g = 0.3 mm, h = 3 mm, Φ_a_ = 220°. **b** Side view of the meta-atom. **c** Transmission amplitude and phase responses of the designed meta-atom as Φ_b_ varies from 10° to 200°. **d** Eight mold-inspired meta-atoms with 3-bit quantized phase values Φ_b_

Extending the movable-type printing to metasurfaces, we design eight categories of meta-atoms as the modular “molds”, thus improving the reusability and feasibility of mechanically reconfigurable metasurfaces. Specifically, we design a high-transmission-efficiency Huygens meta-atom, whose structure is shown in Fig. 2a, b. The proposed meta-atom comprises vertically symmetric metallic layers and a dielectric substrate. The relative permittivity *ϵ*_r_ is set to 2.2, and the loss tangent tan *δ* is 0.001. As the value of Φ_b_ varies, the EM response of the designed meta-atom changes accordingly at the operating frequency. As illustrated in Fig. 2c, when Φ_b_ increases from 10° to 200°, the meta-atom achieves a dynamic phase modulation range approaching 360°, with a transmission amplitude consistently above 0.9. Leveraging this characteristic, we select eight distinct Φ_b_ values to design a set of 3-bit meta-atoms, each is capable of generating a unique EM response and serving as the fundamentally units of the movable-type coding metasurface. The schematic diagram of the eight meta-atoms is shown in Fig. 2d, with their corresponding Φ_b_ values set to 200°, 123°, 111°, 104°, 98°, 90°, 75°, and 10°, respectively. Additional fabrication details are provided in the first section of Materials and Methods.

### Multi-layer movable-type coding metasurfaces for EM computing

The proposed modular meta-atoms can be assembled into a multilayer metasurface architecture, thereby constructing a reconfigurable movable-type DNN for EM computing. The proposed MT-RDNN comprises five layers: an input layer, three reconfigurable hidden layers, and an output layer, with adjacent layers separated by 120 mm (approximately 5.6λ at 14 GHz). The input layer is a metal sheet with hollow regions, designed to tailor the incident wavefront for efficient excitation of the subsequent metasurface layers. The hidden layers are composed of three movable-type coding metasurfaces, each containing 20 × 20 modular meta-atoms. During forward propagation, the incident EM wave first passes through the input layer and is sequentially modulated by the three hidden layers before reaching the output layer. During the backpropagation process, the mean squared error (MSE) loss is adopted to measure the discrepancy between the predicted and target outputs. Then, the phase configurations of the network are optimized by iteratively minimizing the MSE loss (see Supplementary Note 1 for details). Adam optimizer is employed to minimize the loss, thereby enabling the system to perform diverse EM tasks. The impact of modulation precision on computing ability is further discussed in Supplementary Note 2, confirming that the proposed meta-atoms can achieve high recognition accuracy with 3-bit quantization. More detailed model training and derivation are described in the second section of Materials and Methods.

**Fig. 3:**
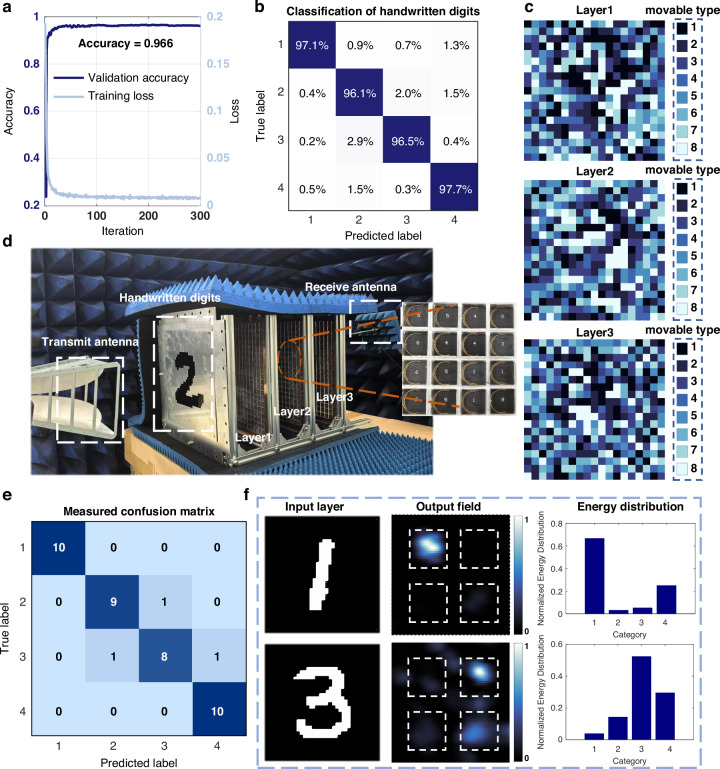
Results of MT-RDNN for handwritten digit classification. **a** Training loss and validation accuracy curves for handwritten digit classification. **b** Confusion matrix on the validation set. **c** Optimized coding patterns of the three hidden layers. **d** Experimental setup of the MT-RDNN for handwritten digit classification. **e** Measured results of the confusion chart for handwritten digits. **f** Classification results and the corresponding electric field distributions

Two classification tasks on the Extended Modified National Institute of Standards and Technology (EMNIST) dataset are performed to evaluate the EM computing capability of the proposed MT-RDNN. The network is initially trained on four categories of digits (“1”, “2”, “3”, and “4”) from the EMNIST dataset. Following data binarization of the training set, the coding patterns of the three metasurface layers in MT-RDNN are optimized using the Adam optimizer. As shown in Fig. 3a, the training loss of the MT-RDNN tends to be stable after approximately 50 iterations. Meanwhile, the validation accuracy converges 96.6% after 300 iterations. Figure 3b displays the confusion matrix on the validation set, indicating that the proposed MT-RDNN achieves a recognition accuracy exceeding 96.1% for the four digital categories. We further find that the recognition accuracy for the 10-class classification task is predominantly determined by the physical capacity of the current architecture, including the number of meta-atoms, the precision of phase modulation, and the number of layers, rather than being restricted by optimization strategies such as the selection of loss function or hyperparameter settings (see Supplementary Note 3 and Table S1 for more details).

The optimized coding patterns for the three hidden layers are shown in Fig. 3c, where each color represents a distinct category of meta-atom. These optimized coding patterns are subsequently implemented to construct the MT-RDNN for handwritten digit classification. The experimental setup is illustrated in Fig. 3d, where a linearly polarized horn antenna is positioned in front of the metallic input layer. Then, the linearly polarized EM wave transmits through the hollow region of the input layer, subsequently impinging on the first metasurface layer. After being modulated by the three hidden layers, the EM wave is focused on the output layer. A scanning near-field microwave microscope (SNMM), positioned 120 mm from the third hidden layer, is utilized to collect the EM wave arrived at the output layer. In Supplementary Note 4, we present the measured transmission efficiency and amplitude responses of 3-bit meta-atoms, which were then incorporated into the network for testing. These results further validate the network’s adaptability and generalization across a wide range of classification tasks. In the experiments, ten samples were randomly selected from each digit category in the test set, resulting in the fabrication of forty metallic input layers. The classification results for the forty samples are summarized in the confusion matrix shown in Fig. 3e, indicating an overall accuracy of 92.5%. Figure 3f shows the results of the MT-RDNN for classifying the handwritten digits “1” and “3”. It could be observed that, the EM wave is concentrated on the upper-left corner of the output layer when digit “1” is input. Meanwhile, the maximum energy appears in the upper-right corner for digit “3”. Furthermore, the electric field distribution across the output layer is quantified by dividing the layer into four regions and independently summing the field intensity within each region. The qualified energy distribution in Fig. 3f also confirms that the region with the highest energy matches the input category. Additionally, the experimental results are consistent with the simulated results, indicating the effectiveness of the proposed MT-RDNN. Furthermore, the impact of alignment errors on the network performance when assembling the MT-RDNN is investigated in Supplementary Note 5.

**Fig. 4:**
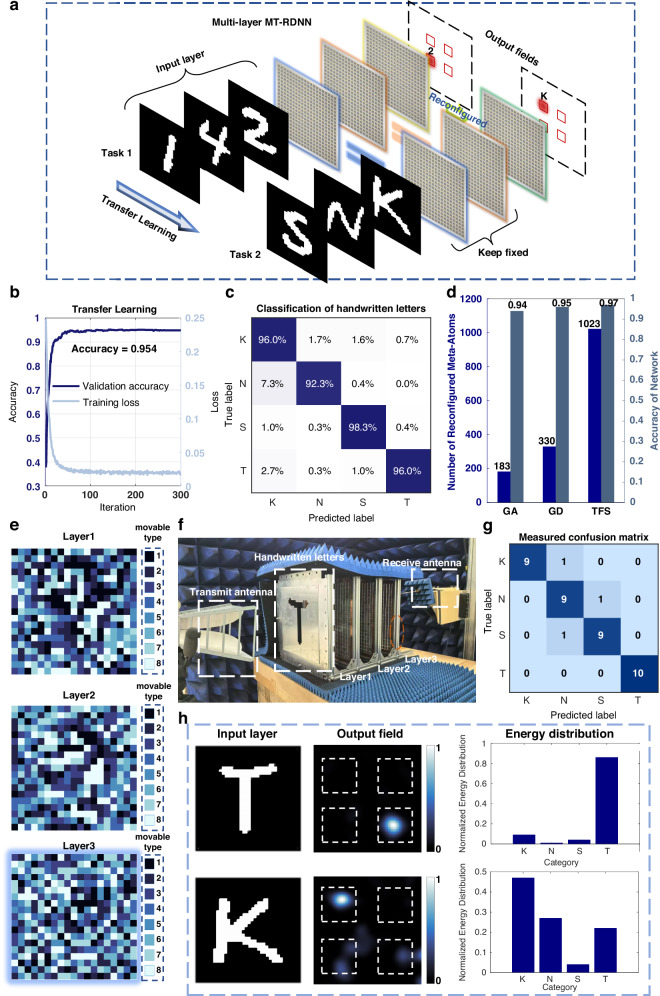
Results of MT-RDNN for task adaptation. **a** Conceptual illustration of task adaptation from handwritten digit to letter classification using transfer learning. **b** Training loss and validation accuracy curves for the English letter recognition task, obtained by fine-tuning the third hidden layer of the MT-RDNN. **c** Confusion matrix of the MT-RDNN fine-tuned with the GD-based transfer learning. **d** Performance comparison of the GA, GD, and TFS algorithms in terms of recognition accuracy and number of reconfigured meta-atoms. **e** Coding patterns of the three hidden layers were fine-tuned with the GD-based transfer learning. **f** Experimental setup of the MT-RDNN for English letter classification. **g** Measured results of the confusion chart for handwritten letter. **h** Experimental classification results and the corresponding electric field distributions

The reconfigurability and task adaptability of MT-RDNN are further evaluated. As shown in Fig. 4a, MT-RDNN trained on the handwritten digit dataset can be adapted to the English letter classification task by updating the coding pattern of the final hidden layer in the network. Specifically, four categories of handwritten English letters, i.e., “K”, “N”, “S”, and “T”, are selected for experiments. To achieve this adaptation efficiently, we employ physical-layer transfer learning, in which certain portions of the coding pattern are retained while the remaining elements are optimized, enabling the implementation of two distinct adaptation strategies. The final hidden layer is fine-tuned using a gradient descent (GD)-based transfer learning algorithm, while the parameters of the other two hidden layers are retained from those of the previously trained digit classification network. The training loss and validation accuracy curves are presented in Fig. 4b, showing that the model achieves an accuracy of 95.4% after approximately 300 iterations. The confusion matrix for the English letter classification task is shown in Fig. 4c. Further classification results obtained by fine-tuning other hidden metasurface layers via the GD algorithm are discussed in Supplementary Note 6. Furthermore, we compare the task adaptation performance of the GD algorithm with that of the genetic algorithm (GA), which is a heuristic transfer learning approach described in Supplementary Note 7. Figure 4d illustrates the performance of the three optimization strategies: GD, GA, and training from scratch (TFS). It should be noted that TFS refers to optimizing the entire coding pattern of the MT-RDNN from scratch, without relying on any pre-trained layers. The simulation results in Fig. 4d indicate that both GD and GA significantly reduce the number of meta-atoms to be reconfigured compared to the TFS strategy, while still maintaining high recognition accuracy. However, although GA requires fewer meta-atom reconfigurations, it leads to a slight degradation in recognition accuracy. Thus, balancing the reconfiguration efficiency and recognition performance, we select the GD-based transfer learning strategy for the subsequent experiments.

The meta-atoms of the third hidden metasurface layer are reconfigured based on the optimization results in Fig. 4e, while the remaining hidden layers are retained from the handwritten digit classification experiment. The experimental setup of the MT-RDNN for English letter classification is shown in Fig. 4f, which is largely consistent with that used for digit classification. Ten samples from each of the four categories were randomly selected and fabricated into a total of forty metallic input layers. The confusion matrix in Fig. 4g summarizes the experimental results, revealing an overall recognition accuracy of 92.5%. Experimental results for classifying the letters “T” and “K” are shown in Fig. 4h. Specifically, when the letter “T” is input, the electric field output by the last hidden layer is predominantly focused in the lower-right region. In contrast, the energy of the incident EM wave is concentrated in the upper-left region when the MT-RDNN is adopted to classify the letter “K”. Moreover, the output layer is evenly divided into four regions to quantitatively evaluate the output electric field distribution. The resulting charts of energy distribution in Fig. 4h reveal the high recognition accuracy of the MT-RDNN in the English letter recognition task. Experimental results demonstrate that the proposed MT-RDNN enables efficient adaptation across different tasks by replacing only a subset of meta-atoms within the multilayer metasurfaces. These results demonstrate that MT-RDNN can efficiently adapt to different tasks by reconfiguring only a subset of meta-atoms while preserving the remaining layers. To further validate its scalability and applicability, we extended the study to more complex scenarios, including ten-class classification as detailed in Supplementary Note 8, and to operating bandwidth analysis as presented in Supplementary Note 9, where experimental results showed an effective bandwidth of approximately 520 MHz. Moreover, the modular and detachable nature of the meta-atoms enables low-cost and seamless task migration, highlighting the potential of MT-RDNN as a practical and scalable solution for EM computing.

### Single-layer movable-type coding metasurface for EM holography

**Fig. 5:**
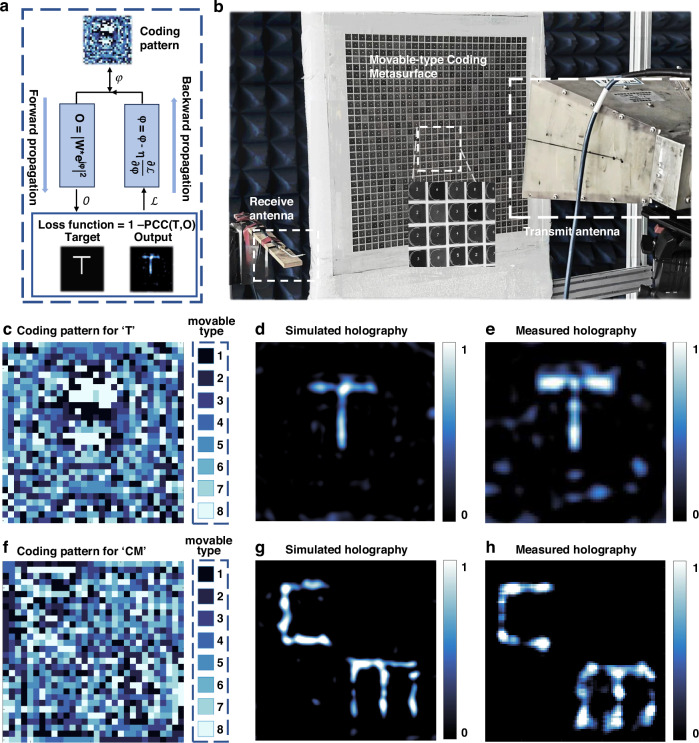
Single-layer movable-type coding metasurface for the EM holography. **a** Flowchart of the GD-based optimization for EM holography. **b** Experimental setup for EM holography. **c** Optimized coding pattern for generating the holographic image “T”. **d**, **e** Simulated and measured holographic reconstructions for “T”. **f** Optimized coding pattern for the holographic image “CM”. **g**, **h** Simulated and measured holographic reconstructions for “CM”

In addition to performing EM computation through a multi-layer architecture, the movable-type coding metasurface can also achieve EM holography using a single-layer configuration. A holography optimization algorithm based on GD^53^ is employed to optimize the coding pattern of the metasurface, as shown in Fig. 5a. Under the assumption of plane-wave incidence, the diffraction between the excitation source and the metasurface can be neglected. Therefore, the forward propagation of the EM wave is expressed as

1 $$O={\left|{\rm{U}}\right|}^{2}={{|W}* {M|}}^{2}={\left|W* {diag}\left({e}^{j\varPhi }\right)\right|}^{2}$$

where $U$ denotes the output EM field reaching the observation plane, and *W* represents the diffraction matrix derived from the Fresnel diffraction approximation. $\varPhi$ denotes the coding pattern matrix of the metasurface, and *O* is the output EM intensity. M =${diag}\left({e}^{j\varPhi }\right)$ denotes a diagonal modulation matrix, where each diagonal element corresponds to the complex transmission coefficient (with meta-atom amplitude and phase $\varPhi$) of a meta-atom in the metasurface. To quantitatively evaluate the hologram quality, the Pearson Correlation Coefficient (PCC) is employed to measure the similarity between the simulated output field distribution and the target pattern, which is calculated as

2 $${PCC}\left(T,O\right)=\,\frac{\sum \left(O-\bar{O}\right)\left(T-\bar{T}\right)}{\sqrt{\sum {\left. (O-\bar{O}\right)}^{2}}\sqrt{\sum {\left(T-\bar{T}\right)}^{2}}}$$

where *T* and *O* denote the target and output EM field distributions, respectively. $\bar{T}$ and $\bar{O}$ denote the average values of the target electric field distribution *T* and the output field distribution *O*, respectively. The PCC quantifies the similarity between the simulated and target patterns, with the value closer to 1 indicating higher similarity. Accordingly, the loss function for optimizing the coding pattern of the metasurface is defined as ${Loss}(T,O)\,=\,1-{PCC}(T,O)$. The gradient of the phase distribution with respect to the loss function is expressed as

3 $$\frac{\partial L}{\partial \varPhi }=\,-2{Im}\left\{M\odot \left({W}^{T}* \left(\frac{\partial L}{\partial O}\odot {U}^{* }\right)\right)\right\}=2{Im}\left\{{M}\odot \left({W}^{T}* \left(\frac{\partial {PCC}}{\partial O}\odot {U}^{* }\right)\right)\right\}$$

where the symbol ⊙ denotes element-wise multiplication, *Im*(·) indicates imaginary part of a complex-valued function, U^*^ represents the complex conjugate of *U* and *W*^*T*^ represents the transposed diffraction weight matrix used for backward propagation, in the direction opposite to *W*.

In the experiment, 900 modular meta-atoms were employed to construct a 30 × 30 movable-type single-layer metasurface with overall dimensions of 420 mm × 420 mm. The experimental setup for EM holography is illustrated in Fig. 5b, comprising the movable-type coding metasurface, a transmitting horn antenna, and a receiving antenna positioned 300 mm behind the metasurface. Two coding patterns of the metasurface, shown in Fig. 5c, f, respectively, were optimized to generate the holographic images “T” and “CM,” respectively. In the experiment, the horn antenna was positioned 2.0 m in front of the metasurface, resulting in an incident wavefront that deviates from the ideal plane-wave assumption. To mitigate this effect, a phase compensation approach was employed, in which the coding pattern obtained from gradient descent optimization was further modified by incorporating the measured phase deviation. Additional experimental configurations under different incident conditions, together with the corresponding compensation strategies, are provided in Supplementary Note 10. Figure 5d, e present the simulated and measured reconstructions of “T”, respectively, indicating that the movable-type coding metasurface can produce a desired holographic image. Similarly, Fig. 5g, h display the simulated and measured field distributions corresponding to the coding pattern in Fig. 5f, showing excellent agreement between simulations and experiments.

### Single-layer movable-type coding metasurface for human vital sign sensing

**Fig. 6:**
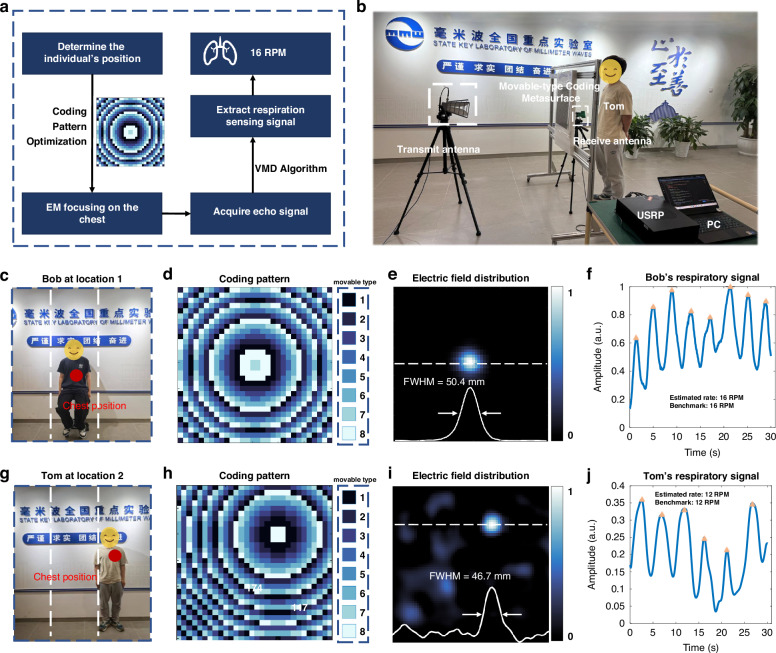
Single-layer movable-type coding metasurface for human vital sign sensing. **a** Flowchart of respiration monitoring using the proposed metasurface-based sensing system. **b** Experimental setup for contactless vital sign sensing. **c** Photograph of vital sign monitoring for Bob at Location 1. **d**, **e** Optimized coding pattern and corresponding electric field distribution when focusing EM wave on Bob’s chest. **f** Respiratory signal of Bob extracted by the sensing system. **g** Photograph of vital sign monitoring for Tom at Location 2. **h**, **i** Optimized coding pattern and corresponding electric field distribution when focusing EM wave on Tom’s chest. **j** Extracted respiratory signal of Tom

Owing to its reconfigurability and EM wave focusing capability, the proposed movable-type coding metasurface can also be employed for contactless human vital sign sensing. This sensing paradigm relies on the analysis of wireless signal perturbations induced by subtle physiological movements, such as respiration-driven chest displacement and skin vibrations. To achieve this, the proposed metasurface focuses EM waves onto the human chest, thereby reducing noise interference from the surrounding environment and limb movements during vital sign sensing. In this way, the sensing system enables the inference of underlying physiological states and behaviors by extracting and interpreting variations in the reflected EM signals. Subsequently, variational mode decomposition (VMD) algorithm^20^ is adopted to extract human respiration signal and estimate the respiration rate. The working pipeline of the respiration sensing system is illustrated in Fig. 6a.

The experimental setup for contactless vital sign sensing is shown in Fig. 6b. A universal software radio peripheral (USRP), controlled via LabVIEW 2019, functions as both transmitter and receiver. A 3.5 GHz baseband signal is generated and upconverted to 14 GHz using a frequency mixer, then transmitted toward the metasurface through a horn antenna. The signal reflected from the human chest is captured by a receiving antenna and down converted using the USRP. Meanwhile, a wearable piezoelectric sensor HKH-11C is employed as the reference to acquire ground-truth respiratory data. Both the USRP and HKH-11C are synchronized via the timestamps to ensure precise temporal alignment. Figure 6c, g illustrate the vital sign monitoring results of Bob and Tom, respectively, both located approximately 300 mm from the metasurface. Owing to their distinct spatial positions, two distinct coding patterns were optimized to focus the transmitted energy onto the chest regions of each subject, as shown in Fig. 6d, h. Then, the measured EM field distributions are presented in Fig. 6e, i, respectively. The full width at half maximum (FWHM) is employed to quantify the focal spot size, as indicated by the white dashed lines in Fig. 6e, i. Specifically, the FWHM of the two focal spots are measured to be 50.4 mm and 46.7 mm, respectively, confirming efficient energy concentration on the targeted chest regions. The extracted respiratory signals of Bob and Tom, shown in Fig. 6f, j, exhibit clear periodicity, enabling accurate estimation of respiration rates consistent with benchmark sensor measurements. Furthermore, Supplementary Note 12 presents additional discussions about the effects of mutual interference in multi-subject scenarios, as well as variations arising from different clothing conditions. These results collectively demonstrate the effectiveness of the single-layer movable-type coding metasurface for contactless sensing, highlighting its potential for non-invasive and real-time monitoring of human vital signs.

## Discussion

Conventional DNNs typically lack reconfiguration and task adaptation capabilities, which constrain their flexibility and scalability for EM computing. In this work, we proposed an MT-RDNN composed of multiple movable-type coding metasurfaces, which enabled efficient task adaptation through modular meta-atom reassembly, thus providing a versatile EM computing platform with post-fabrication reconfigurable capabilities. Experimental results demonstrated that the MT-RDNN could be transferred from the handwritten digit recognition task to the English letter classification task by reconfiguring only the final hidden metasurface layer, achieving an accuracy exceeding 92% in both tasks. These findings underscore the task adaptability, modular scalability, and practical potential of the MT-RDNN for EM computing.

Compared with conventional reconfigurable DNNs, such as electronically or thermally tunable optical networks and mechanically reconfigurable metasurface networks, the MT-RDNN demonstrates remarkable flexibility, scalability, and practical applicability. Although mechanical reconfiguration operates at lower switching speeds, the modular and unit-level meta-atom design combined with physical-layer transfer learning allows the network to adapt to new tasks by fine-tuning only a subset of meta-atoms. Therefore, both the computational and physical reconfiguration overheads can be substantially reduced. For a 20×20 three-layer MT-RDNN, full-network optimization requires tuning approximately 1,200 parameters and replacing over 1000 meta-atoms, with a training time of 14.6 seconds and a physical replacement time of about 70 minutes. In contrast, only the final layer requires adjustment when applying transfer learning to the MT-RDNN for network optimization, which consists of around 300 meta-atoms. This approach reduces the training time to approximately 12.2 seconds and decreases the physical reconfiguration time to 20 minutes, thereby cutting the overall operational time by more than two-thirds. These results indicate that, despite the inherent speed limitations of mechanical control, the MT-RDNN enables scalable, low-cost, and multifunctional reconfiguration with reasonable time and labor requirements, highlighting its practical potential for task-adaptive EM computing.

Further experiments were conducted to verify the feasibility and effectiveness of the single-layer movable-type coding metasurface for EM holography and contactless human vital sign sensing. The results validated the capability of the proposed metasurface for high-fidelity holography and accurate respiration monitoring of multiple individuals. The experiments across EM computing, holography, and vital sign sensing highlighted the functional versatility, scalability, and practical applicability of the MT-RDNN and movable-type coding metasurfaces. In other words, the modular design and efficient reconfigurability enable the realization of low-cost, scalable, and reprogrammable microwave DNNs. Moreover, this work opened new opportunities to advance task-adaptive multifunctionality by incorporating hybrid control mechanisms (e.g., combining mechanical reconfiguration with electrical or optical tuning), thereby reducing reconfiguration overhead and enabling seamless integration of computational, holographic, and sensing tasks. Such progress could ultimately enable real-time, intelligent metasurface systems for emerging applications in wireless communications, human-machine interaction, and healthcare monitoring.

## Materials and methods

### Fabrication of the moveable-type coding metasurface

The modular meta-atoms were fabricated using printed circuit board (PCB) technology. Square dielectric spacers were made from 3-mm-thick F4B material with a relative permittivity of 2.2 and a loss tangent of 0.001. The copper surfaces were treated with electroless nickel immersion gold (ENIG) to prevent oxidation and mechanical wear. Each meta-atom was produced with an edge tolerance of approximately ±0.1 mm to ensure dimensional uniformity. Furthermore, the durability of meta-atoms was verified by performing more than 300 insertion and removal cycles without detectable physical damage or performance degradation. For experimental demonstrations, 400 modular meta-atoms per quantization level were fabricated in the 3-bit set. The meta-atoms were mounted on an acrylic platform with a uniform inter-element spacing of 2 mm in each layer. In the MT-RDNN configuration, each layer consisted of a 20 × 20 array of meta-atoms, while the single-layer movable-type metasurface employed a 30 × 30 array for the tasks such as EM holography and vital sign sensing.

In the MT-RDNN design, the input layer was constructed from the binarized EMNIST dataset, where the pixel value of “1” denoted a hollow (transmissive) region and “0” denoted a metallic (reflective) region. Each pixel was defined with a size of 7 mm, forming a 28 × 28 matrix. Therefore, the corresponding metallic mask measured 196 mm × 196 mm with a thickness of 1 mm. The mask layer was fabricated from aluminum alloy 5052, which has a typical electrical conductivity of ~2.0 × 10^7 ^S/m. At the operating frequency range, the skin depth of this material is on the micrometer scale, much smaller than the mask thickness. Consequently, the metallic regions effectively reflect EM waves, while the hollow regions permit EM wave transmission, thereby realizing binary input modulation.

### Training of the MT-RDNN

Our MT-RDNN was trained using Python 3.7.0 and the TensorFlow 2.4.1 framework (Google Inc.) on a desktop computer equipped with an Intel(R) Core (TM) i5-10500 CPU @ 3.10 GHz, 32 GB RAM, and the Microsoft Windows 10 operating system. The mean square error (MSE) was adopted as the loss function, and the Adam optimizer was used to update the phase values of each network layer. For the digit recognition task, 6000 training and 1000 validation samples were used per class with a batch size of 4000. For the letter recognition task, 2400 training and 300 validating samples were used per class with a batch size of 2400. Both datasets were trained for 50 epochs with a learning rate of 0.1.

## Supplementary information

SUPPLEMENTARY MATERIAL

## Data Availability

The data that support the findings of this study are available from the corresponding author upon request.
