# Supplementary material for: Multifunctional movable-type coding metasurface enabling reconfigurable diffractive neural networks
Source: Light Sci Appl. 2026 Feb 26;15:127. doi: 10.1038/s41377-026-02216-6 (PMC12946256; doi:10.1038/s41377-026-02216-6)
Supplement: Supplementary file 1 — SUPPLEMENTARY MATERIAL [file 41377_2026_2216_MOESM1_ESM.pdf]

Supplementary Information for

Multifunctional Movable-Type Coding Metasurface Enabling Reconfigurable Diffractive Neural Networks

Zhicai Yu[1,2,3], *Xinyu Li*[1,2,3], *Ze Gu*[1,2], *Long Chen*[1,2], *Jianlin Su*[1,2], *Zixuan Cai*[1,2], *Xinyi Yu*[1,2], *Shilong Qin*[1,2], *Lei Zhang*[1,2], *Qian Ma*[1,2,*], *Jian Wei You* [1,2,*] *and Tie Jun Cui*[1,2,*]

[1] State Key Laboratory of Millimeter Wave, Southeast University, Nanjing 210096, China

[2] Institute of Electromagnetic Space, Southeast University, Nanjing 210096, China

[3] These authors contributed equally: Zhicai Yu, Xinyu Li.

*Corresponding authors: maqian@seu.edu.cn, jvyou@seu.edu.cn, tjcui@seu.edu.cn

Supplementary Note 1. Computational model for diffractive neural network

Assuming the electromagnetic wave passing through the input layer is I_0, the interlayer diffraction is governed by the Rayleigh-Sommerfeld diffraction theory,

$$W_i^k(x,y,z) = \frac{z-z_i}{r^2}\left(\frac{1}{2\pi r}+\frac{1}{j\lambda}\right)\exp\left(\frac{j2\pi r}{\lambda}\right) \tag{S1}$$

where λ denotes the working wavelength of the incident light; r represents the propagation distance of the wavefront.

Considering both accuracy and physical size, we designed the network architecture using three cascaded metasurfaces. The forward propagation model of the entire diffractive neural network can be derived as follows:

$$O = |U_{N+1}|^2 = \left|W_{\mathrm{N}+1}\left(\prod_{k=N}^{1} M_k W_k\right) I_0\right|^2 \tag{S2}$$

where W_k denotes the diffraction weight matrix for the propagation of light from the $(k-1)^{th}$ layer to the kth layer; $M_k = diag\left(e^{j\Phi_k}\right)$ indicates the diagonalization of the vector diffraction modulation at the kth layer with phase coefficients Φ_k. U_{N+1} denotes the output EM field reaching the observation plane. Let *L (O, T)* denote the loss function of the DNN that measures the difference between the network output O and the ground-truth label T. In this case, we use the mean squared error (MSE) as the loss function, i.e., $L(O,T) = \left|\left|O-T\right|\right|_2^2$, where$|| \, ||_2^2$ denotes the L_2 norm. The gradient of the defined loss function with respect to the phase modulation coefficients Φ_k of the kth layer can be derived as

$$\frac{\partial L}{\partial \Phi_k} = \frac{\partial L}{\partial \mathrm{O}}\frac{\partial O}{\partial U_{N+1}}\frac{\partial U_{N+1}}{\partial \Phi_k}$$
$$= -2Im\left\{\left(\left(\prod_{i=k}^{1} M_i W_i\right) I_0\right)\odot\left(\left(\prod_{i=k+1}^{\mathrm{N}} W_i^T M_i\right) W_{\mathrm{N}+1}^T\left(\frac{\partial L}{\partial \mathrm{O}}\odot U_{N+1}^*\right)\right)\right\} \tag{S3}$$

where $\odot$ denotes element-wise multiplication, and $Im(:)$ indicates the imaginary part operator. U_{N+1}^* represents the complex conjugate of U_{N+1}, with the gradient defined as $\frac{\partial L}{\partial \mathrm{O}} = 2(O-T)$. According to the reciprocity principle of light propagation, W_k^T represents the diffraction weight matrix for backward propagation in the opposite direction of W_k between $(k-1)^{th}$ layer and k^{th} layer.

Supplementary Note 2. Influence of modulation precision on MT-RDNN performance

To evaluate the effect of modulation precision on the MT-RDNN, classification accuracy was systematically assessed under discretization levels of 5, 4, 3, 2, and 1 bit, as shown in Figs. S1a-e. The aggregated results in Fig. S1f indicate that accuracy remains largely invariant at resolutions of 8 bits or higher (loss ~0.4%), whereas coarse modulation leads to a pronounced degradation, decreasing to 90.5% at 1 bit. In this representative classification scenario, the 3-bit configuration maintains high accuracy while substantially reducing implementation complexity, exemplifying a practical trade-off between computational performance and hardware feasibility. These observations further support the adoption of 3-bit modulation precision in our experimental implementation.

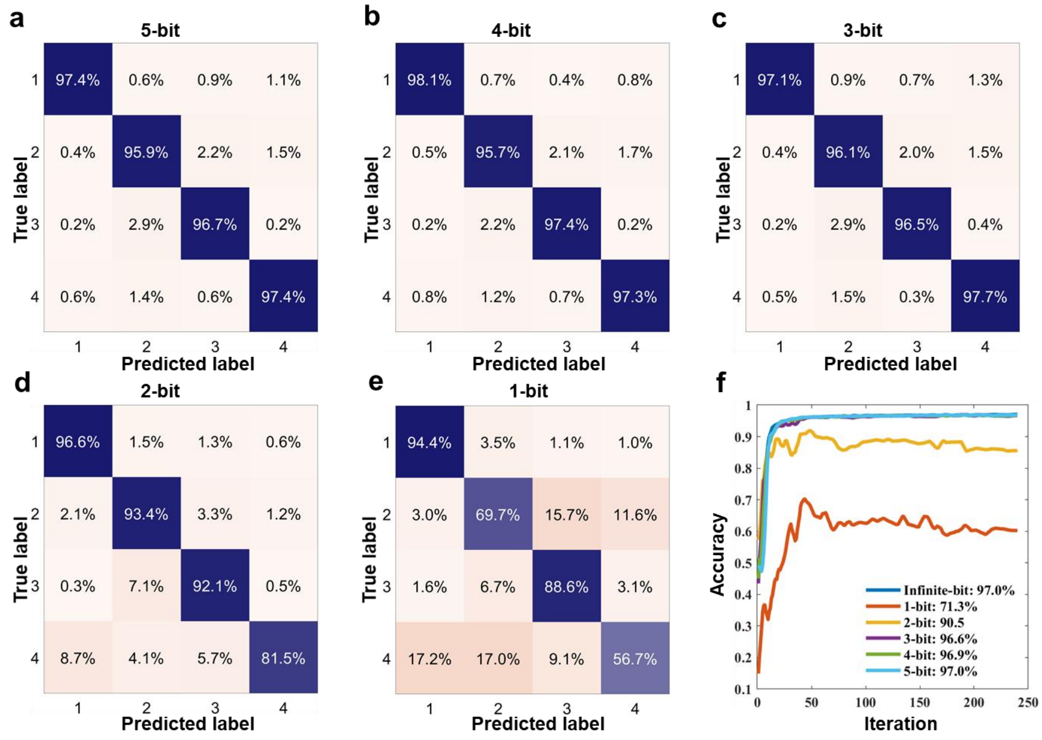

Fig. S1 | Confusion matrices at various phase quantization levels. a 5-bit quantization. **b** 4-bit quantization. **c** 3-bit quantization. **d** 2-bit quantization. **e** 1-bit quantization. **f** Classification accuracy comparison across quantization levels.

Supplementary Note 3. Discussions on the accuracy of classifying more categories

As described in the main text, the MT-RDNN was first evaluated on a four-class classification task. To further assess the scalability of the proposed MT-RDNN, we conducted experiments on the ten-class MNIST recognition task, using the same three-layer configuration with 20×20 meta-atoms per layer. Fig. S2 presents the corresponding classification results under 3-bit phase quantization. It is observed from Figs. S2a-b that the network converged after approximately 300 iterations and achieved an accuracy of 88.2%, showing a moderate performance drop relative to the four-class task due to the increased complexity. We further explored the influence of different optimization strategies, as shown in Fig. S2c by comparing Adam and stochastic gradient descent (SGD). While SGD converged more slowly than Adam, the final accuracy was only 1.4% lower than Adam's. In addition, the impact of network depth and modulation precision on performance was investigated, as illustrated in Fig. S2d. The results showed that increasing the number of layers could improve recognition accuracy for the ten-class task. However, the improvement gradually diminished with deeper networks, eventually reaching a plateau. Furthermore, employing 3-bit quantization on the MT-RDNN resulted in a slight accuracy reduction, with performance approximately 2% lower than that of the unquantized network.

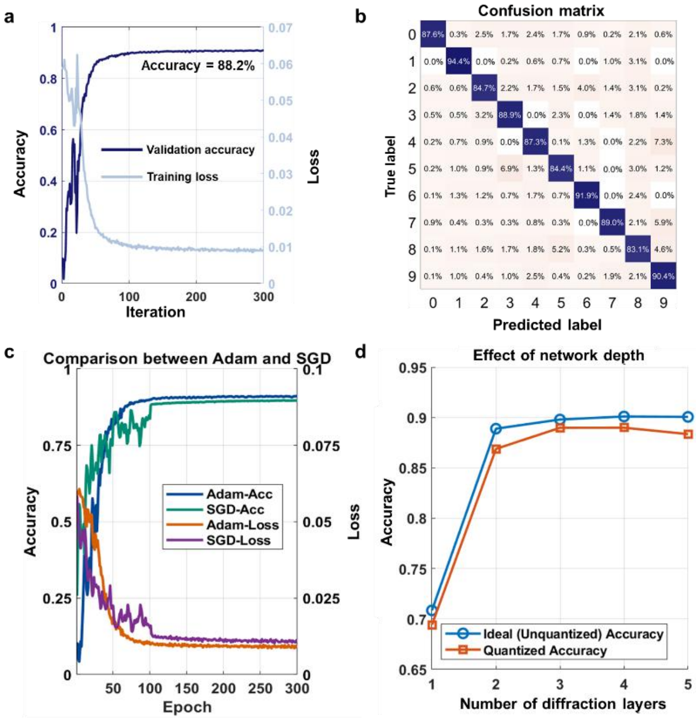

Fig. S2 | Classification results for the ten-class digit recognition task. **a** Training loss and validation accuracy curves. **b** Confusion matrix on the validation set. **c** Comparison of network

trained with Adam and SGD optimizers. **d** Effect of network depth and phase quantization on MT-RDNN performance for the ten-class classification task.

Furthermore, Table S1 compares the proposed MT-RDNN with three representative diffractive neural networks, exploring the effects of loss function, optimizer, modulation precision, and the number of meta-atoms on recognition accuracy. The results indicate that variations in loss function or optimizer have minimal impact, whereas increasing the modulation precision and the number of meta-atoms substantially improves performance. As a result, additional meta-atom can be integrated into MT-RDNN to handle more complex tasks owing to the scalable design of the proposed architecture. These observations suggest that the recognition performance of the MT-RDNN is primarily constrained by the physical capacity of the system rather than the choice of optimization strategy.

Table S1. Comparison of classification performance among diffractive neural networks

Relevant Works	Loss Function/ Optimizer	Modulation Precision	Learnable Parameters (Number of Meta-atoms)	Recognition Accuracy
**Our work**	**MSE/Adam**	**3-bit**	**20*20*3**	**88.2% (10 classes)**
[S1]	MSE/Adam	Continuous	200*200*5	91.75% (10 classes)
[S2]	Cross-Entropy/Adam	5-bit	32*32*3	90.0% (9 classes)
[S3]	MSE/SGD	3-bit	200*200*1	77.2% (10 classes)

Supplementary Note 4. Impact of measured amplitude and phase responses on network recognition accuracy

In numerical simulations, the meta-atoms were assumed to exhibit ideal transmission amplitudes. However, the transmission amplitude and phase of the 3-bit quantized meta-atoms exhibit deviations from the simulated results due to fabrication imperfections. Therefore, the transmission properties of the eight fabricated meta-atom categories were measured to further assess the impact of these deviations on network performance, as shown in Fig. S3a. As illustrated in Figs. S3b-c, the measured transmission amplitudes were lower than the simulated values. However, the phase responses remained almost linear and maintain 3-bit quantization. Specifically, the measured phases of the eight states at 14 GHz were 28.85°, 71.56°, 106.50°, 203.42°, 160.09°, 113.87°, 54.65°, and 10.12°. The degradation and non-uniformity of the measured transmission amplitude can be attributed to fabrication tolerances, material absorption, and minor dimensional variations between meta-atoms. Nevertheless, the measured results demonstrated that the phase responses of the eight meta-atom categories covered 2π, with phase error of less than $\pi/10$.

Additionally, to quantitatively assess the accuracy of the modulation layer, we incorporated the measured amplitude responses of the eight meta-atom categories into the optimized coding sequences. In other words, the coding pattern optimization algorithm assumes uniform amplitude across all meta-atoms, whereas we replaced the theoretically identical amplitude responses with the measured ones. Then, the results of classifying the four categories of digits are presented in Figs. S3d-e. The results demonstrated that the MT-RDNN, composed of meta-atoms with measured amplitude responses, achieved an accuracy of 96% for the task, with only a 0.7% decrease compared to the network with theoretical responses. Meanwhile, the MT-RDNN with measured amplitude responses achieved 95% accuracy for the letter recognition task, with only a 1.7% decrease compared to the network with theoretical responses. These results confirm that although practical amplitude losses are unavoidable, the metasurface maintains effective phase modulation, thereby ensuring robust wavefront control and reliable holography.

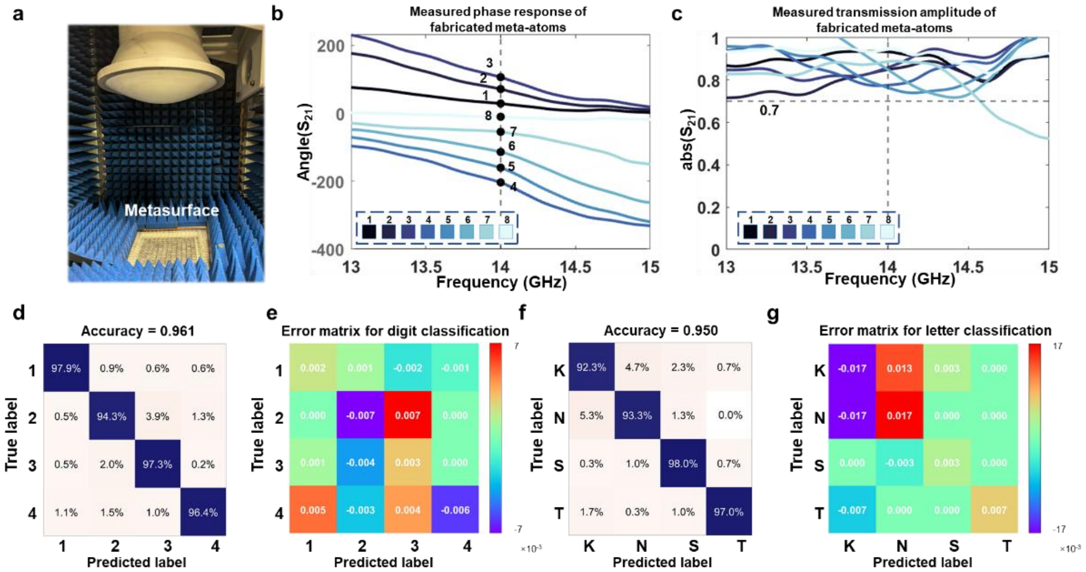

Fig. S3 | Impact of measured amplitude and phase responses on network recognition accuracy. **a** Experimental setup for transmission performance characterization. **b-c** Measured transmission amplitudes and phases of the 3-bit meta-atoms. **d-e** Confusion matrix and error matrix for the digit recognition task when using the measured amplitude and phase responses. **f-g** Confusion matrix and error matrix for the letter recognition task when using the measured amplitude and phase responses.

Supplementary Note 5. The impact of alignment errors on the performance of the MT-RDNN

In this section, we investigate the impact of alignment errors on the performance of the MT-RDNN. Two alignment errors are considered, including intra-unit misalignment and inter-unit misalignment. Specifically, the cutouts on the acrylic plates were designed to hold meta-atoms, ensuring precise alignment of meta-atoms. The fabrication tolerance is approximately ±0.3 mm for the slots and ±0.1 mm for the meta-atom structures, resulting in a potential deviation of each meta-atom from its designed center of roughly ±0.4 mm. Meanwhile, we have customized a metal frame to fix the metasurface layers in MT-RDNN, thereby precisely controlling the relative positions of each layer. Considering both fabrication and assembly tolerances, the horizontal displacement between layers is estimated to be about ±2 mm. The inter-layer spacing is determined by screw positioning holes, with a tolerance of ±0.5 mm, which is negligible compared with the designed 120 mm spacing.

Furthermore, we have conducted 100 random trials on the coding pattern in Figs. 3c and 4e of the main text, and performed an alignment-error analysis. The results in Fig. S4a indicate that the random unit deviations (up to ±0.4 mm) have little impact on network performance. Specifically, the classification accuracy remains approximately 96% for digit classification and 95% for letter classification. In contrast, the performance fluctuates significantly when inter-layer misalignment is introduced. In detail, the average accuracies for digit and letter recognition decreases to 94.3% and 93.6%, respectively. This degradation is mainly attributed to the cumulative phase distortions caused by inter-layer misalignment at the operating frequency of 14 GHz.

To mitigate these effects, two strategies can be employed. First, assembly precision can be improved by using high-accuracy positioning structures, such as 3D-printed components, to limit inter-layer lateral displacement to below 1 mm. Second, incorporating mild random perturbations, such as translations and rotations, into the training dataset can enhance model robustness and effectively suppress performance degradation caused by alignment errors.

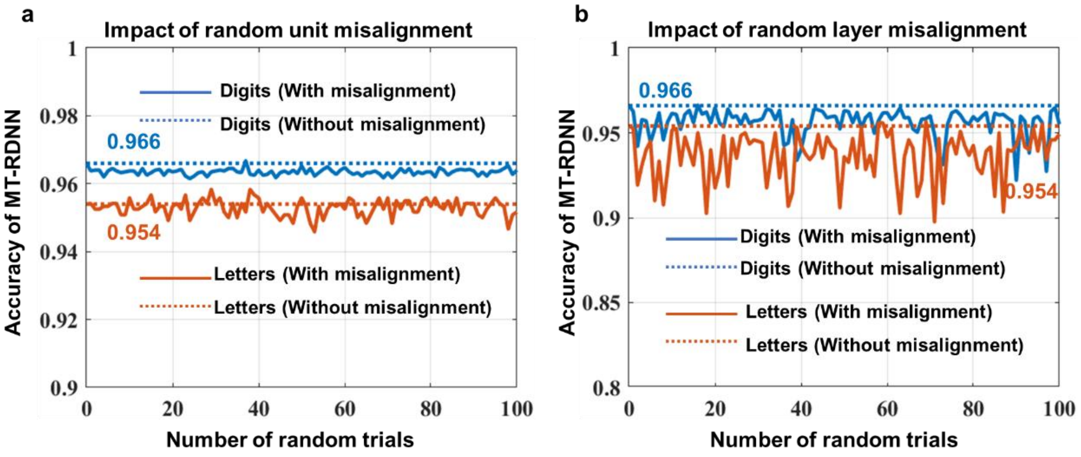

Fig. S4 | The impact of alignment errors on the performance of the MT-RDNN. a Effect of random unit-position deviations on classification accuracy (100 trials). **b** Effect of random inter-layer misalignment on classification accuracy (100 trials).

Supplementary Note 6. Transfer learning algorithm in MT-RDNN

Transfer learning, which enables the application of knowledge acquired from one task to a related but distinct task, was employed to classify handwritten letters using a pre-trained model originally trained on handwritten digits. Phase optimization for each layer was conducted sequentially according to the procedures described in Supplementary Note 1. Specifically, when optimizing the third layer, the phase profiles of the first two layers were held constant, and only the third layer was updated during each iteration (i.e., when $k = 3$ in Equation 3). The same strategy was applied to the other layers individually.

Fig. S5 presents the classification results obtained by modifying a single layer while keeping the remaining two fixed. The overall accuracy ranged from 93% to 95%, depending on which layer was retrained. Notably, re-optimizing the first layer led to a slightly lower accuracy of 93%, whereas modifying the second or third layer individually still resulted in accuracies exceeding 95%. These results confirm that the MT-DNN architecture supports efficient task reconfiguration via partial retraining, highlighting the practical effectiveness of transfer learning in diffractive neural networks.

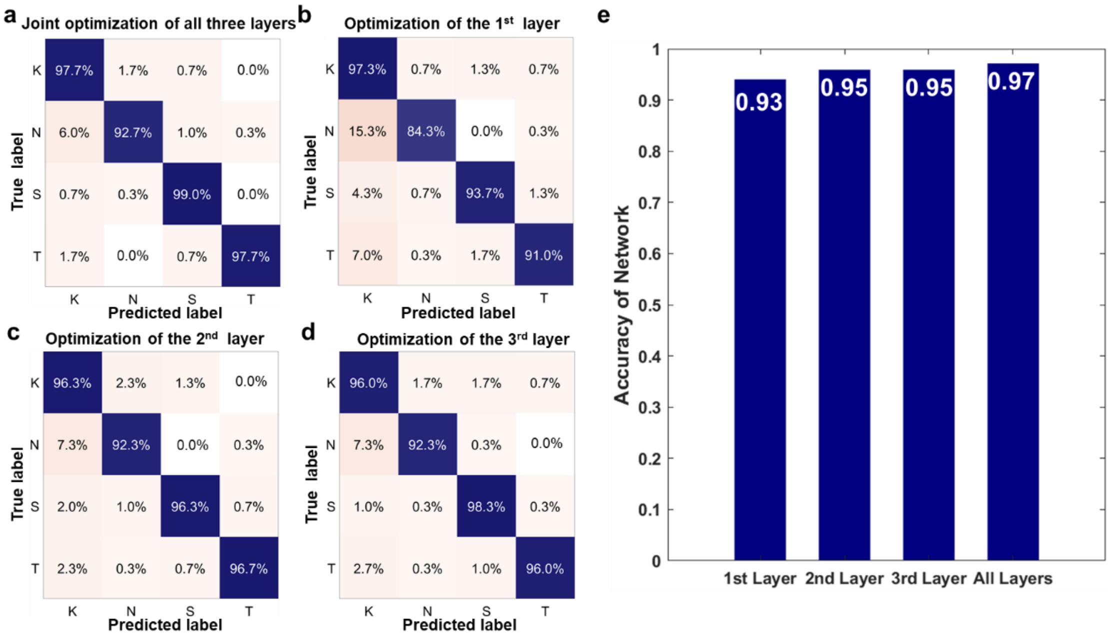

Fig. S5 | Optimizing the impact of different hidden layers on network accuracy. **a** Confusion matrix for altering all three hidden layers simultaneously. **b** Confusion matrix for modifying the first hidden layer. **c** Confusion matrix for modifying the second hidden layer. **d** Confusion matrix for modifying the third hidden layer. **e** Corresponding classification accuracy.

Supplementary Note 7. Implementation strategies for heuristic optimization algorithms

To minimize the degree of meta-atom reconfiguration under the constraint of 3-bit encoding at each lattice site, heuristic optimization techniques were introduced to realize efficient network-to-function transfer. Such approaches employ rule-based strategies to approximate near-optimal solutions within high-dimensional and highly discrete design spaces. In this study, a genetic algorithm (GA) was implemented as the optimization framework. Consistent with the transfer learning strategy discussed earlier, the final hidden layer—comprising 400 meta-atoms, each characterized by 8 discrete phase states—was selected as the optimization target. The fitness function was defined as a weighted combination of classification accuracy and unit retention ratio:

$$Fitness = 0.9 * Accuracy + 0.1 * KeepRate \qquad (S4)$$

where $Accuracy$ denotes the classification performance of the network (as defined in Eq. S2), whereas $KeepRate$ quantifies the proportion of meta-atoms that remain unchanged during reconfiguration.

During the optimization process, a subset of training samples was iteratively fed into the network. Phase configurations that yielded higher fitness were preserved and propagated through standard GA operations, including selection, crossover, and mutation. The optimization process converged after 1,000 iterations, achieving a classification accuracy of 94.6% while maintaining a unit retention ratio of 54.1%, as shown in Fig. S6b.

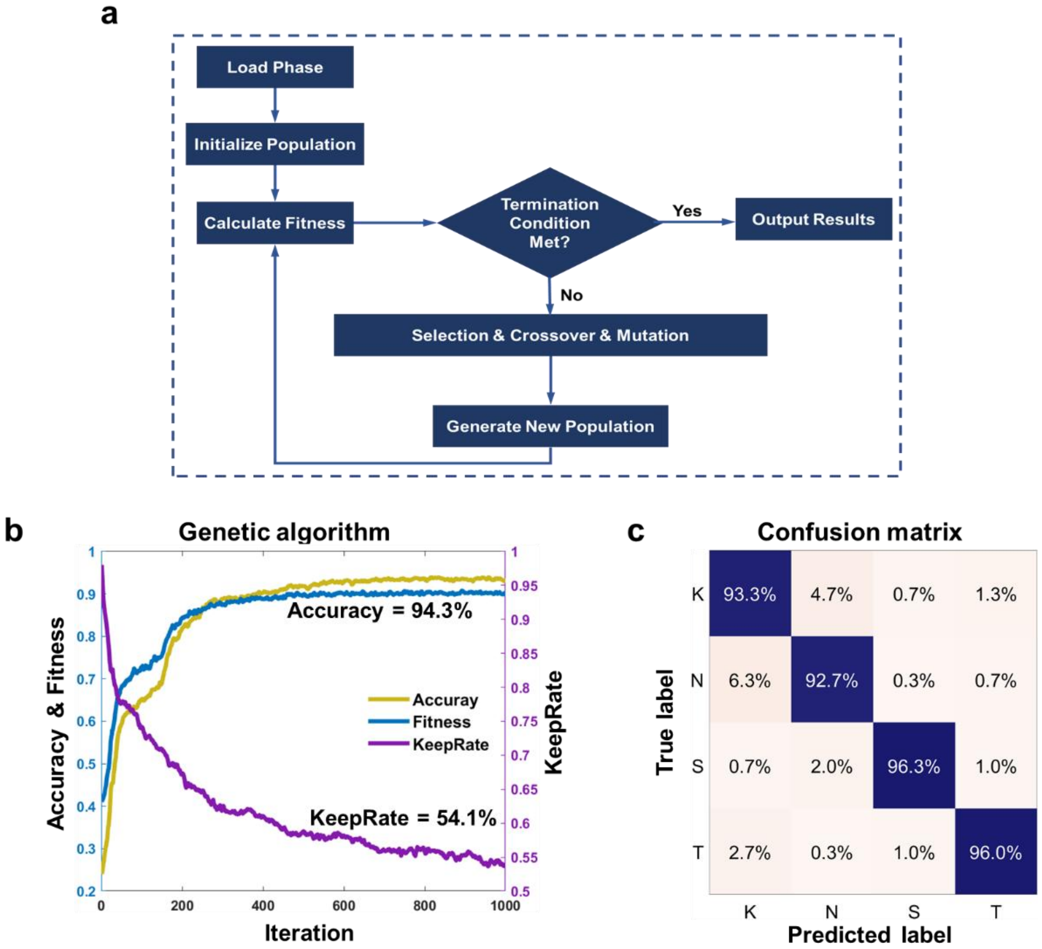

Fig. S6 | Genetic Algorithm for Task Transfer. **a** Flowchart illustrating the genetic optimization procedure. **b** Validation accuracy and unit retention ratio (*KeepRate*) as functions of optimization iterations for handwritten letter classification. **c** Confusion matrix of the validation set after convergence of the optimization process

Supplementary Note 8. Extended verification of transform learning for 10-class classification

In the main text, the MT-RDNN was validated on a four-class classification task to demonstrate its conceptual feasibility. To further evaluate the scalability of MT-RDNN, we applied the same 20×20 three-layer architecture to a ten-class letter classification task. However, simulated results showed that fine-tuning only the last layer provided insufficient optimization depth and led to a noticeable decline in accuracy. To overcome this limitation, both the second and third layers in the MT-RDNN were optimized while the first layer remained fixed. As shown in Fig. S7a, training from scratch achieved an accuracy of 85.5%. In contrast, the accuracy reached 84.8% after applying transfer learning, as shown in Fig. S7b, with only a slight decrease compared to retraining the entire network. Fig. S7c further compares the network performance and the number of reconfigurable meta-atoms between training from scratch and transfer learning, showing that reducing the optimized layers resulted in an accuracy drop of approximately 3%, mainly due to limited phase-control freedom. These results confirm that the MT-RDNN remains effective for the ten-class recognition task. In other words, the architecture supports flexible layer optimization under a fixed scale, and transfer learning helps reduce training and reconfiguration overhead.

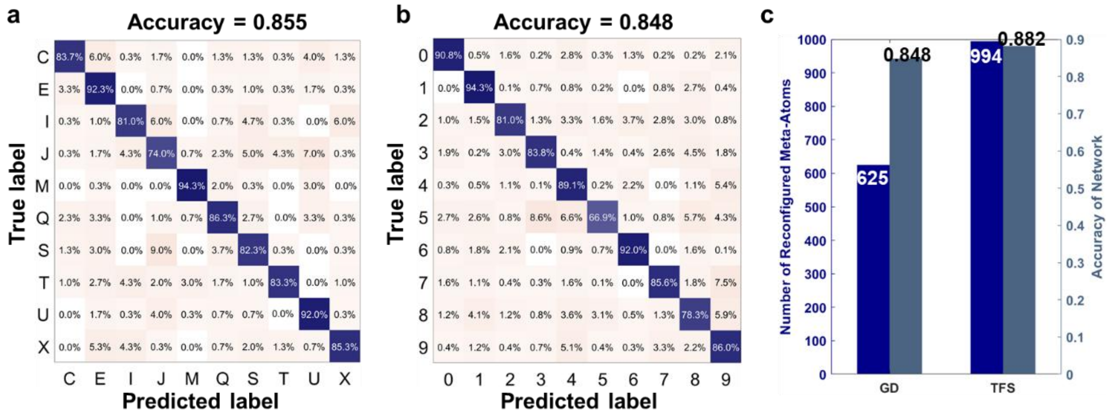

Fig. S7 | Extended verification of MT-RDNN for 10-class classification. a Confusion matrix for the letter classification task with all layers optimized. **b** Confusion matrix for the digit classification task using physical-layer transfer learning, where only the second and third layers are fine-tuned. **c** Performance comparison between training from scratch (TFS) and transfer learning for the digit classification task.

Supplementary Note 9. The operating bandwidth of the MT-RDNN

We evaluated the operating bandwidth of the proposed MT-RDNN by testing all 80 samples with the measured transmission responses of the fabricated meta-atoms, and compared the results with the numerical simulations. For digit recognition, Fig. S8a shows that the accuracy remained above 90% within 13.62-14.45 GHz, corresponding to an effective bandwidth of about 0.83 GHz in the simulated case. When using the measured responses, the working frequency was 13.57-14.19 GHz, with an effective bandwidth of 0.62 GHz. For letter recognition, Fig. S8b shows that the effective bandwidth was about 0.75 GHz in the simulated case. Meanwhile, the measured working bandwidth is approximately 0.52 GHz.

Experimental results demonstrated that the measured bandwidths for the two classification tasks are consistently narrower than the simulated ones, which can be attributed to fabrication tolerances, array misalignment, and electromagnetic interference during measurements. Conservatively, the effective operating bandwidth of the MT-RDNN is estimated to be approximately 0.52 GHz, as determined by the letter recognition task. It is also observed that the effective bandwidth for letter recognition is narrower than that for digit recognition. This reduction is mainly due to that transfer learning requires adapting pretrained weights to a new data distribution, thereby lowering robustness to frequency variations and slightly reducing usable bandwidth.

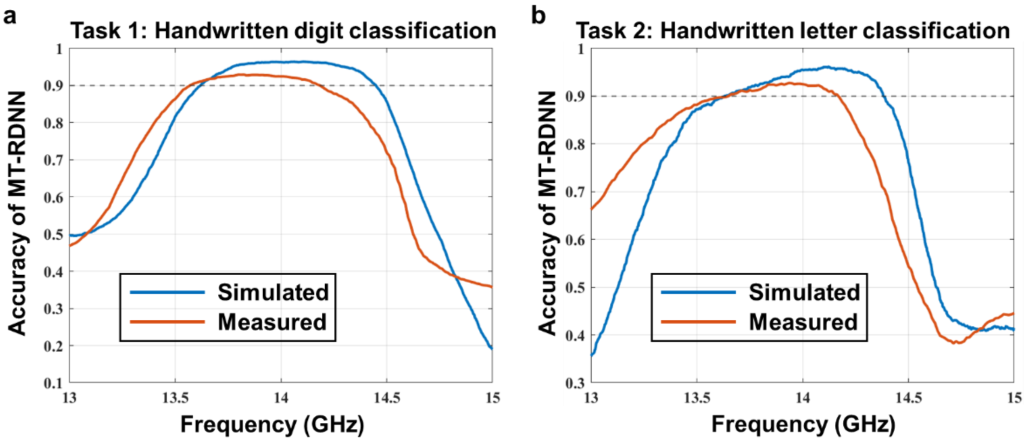

Fig. S8 | The operating bandwidth of the MT-RDNN. a Simulated and measured classification accuracy for handwritten digit classification. b Simulated and measured results for handwritten letter classification after transfer learning

Supplementary Note 10. Influence of non-ideal incident wavefronts on holographic quality

In the main text, the coding patterns were derived under the assumption of ideal plane-wave incidence. However, the incident electromagnetic waves are typically non-ideal spherical waves in practice. To mitigate for the effect of spherical wavefronts on holographic imaging, a phase compensation strategy is implemented. Specifically, the phase compensation approach involves superimposing the additional phase differences induced by the actual incident wavefront onto the pre-optimized coding pattern. In this manner, the impact of spherical wave incidence can be effectively mitigated, resulting in a modulation response closely approximating that under ideal plane-wave excitation.

As illustrated in Fig. S9a, the receiving antenna was positioned 2.0 m in front of the metasurface, and the corresponding incident wavefront was experimentally measured. Within the metasurface aperture, the phase distribution was observed to be non-uniform. Specifically, the central region exhibited relatively uniform phase values, while the peripheral region showed substantial phase deviations from the center, with a maximum difference of approximately 6.2 rad, equivalent to 356°. In this circumstance, the phase compensation mechanism is adopted to incorporate the non-uniform phase distribution into the optimized coding pattern, thus enabling successful reconstruction of the holographic image “T” and “CM”. To further evaluate the versatility of the proposed phase compensation approach, a more challenging scenario with pronounced phase variation was considered. In this case, the receiving antenna was positioned only 0.1 m from the metasurface. Then, the resulting incident wavefront was measured, as shown in Fig. S9c. The wavefront exhibited a spherical profile, with phase values varying markedly across spatial positions. By incorporating this phase distribution into the optimized coding pattern, the corresponding holographic reconstructions were realized, as shown in Fig. S9e.

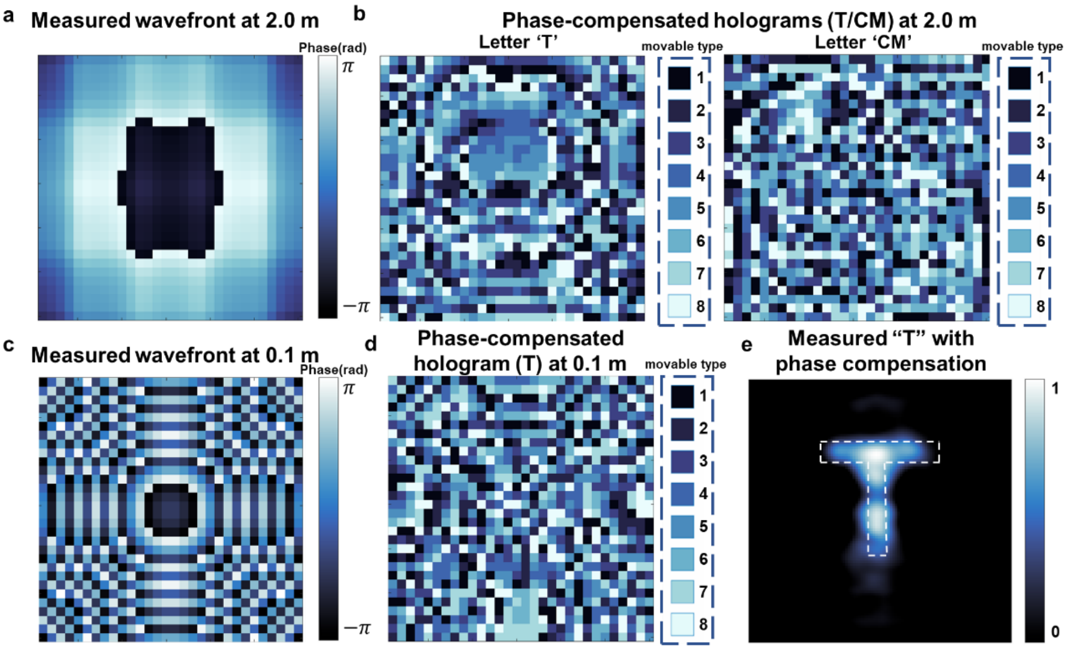

Fig. S9 | Measured incident wavefronts and phase-compensation results. a Measured incident wavefront when the receiving antenna is placed 2.0 m in front of the metasurface. **b** Phase-compensated coding patterns for the holographic image "T" and "CM". **c** Measured incident wavefront when the receiving antenna is placed 1.0 m in front of the metasurface. **d** Phase-compensated coding pattern for the holographic image "T". **e** Measured holographic image "T" using the phase-compensated coding pattern in **d**.

Supplementary Note 11. Effect of imaging distance on holographic reconstruction.

To evaluate the influence of imaging distance on holographic performance, we have conducted both numerical and full-wave simulations for the distances from 0.1 m to 1.0 m. Our metasurface operates at 14 GHz (λ ≈ 21.43 mm) with an aperture of D = 420 mm. Using the common Fresnel-region estimate:

$$z_{Fresnel} \approx \frac{2D^2}{\lambda} \approx 16.5m \qquad (S5)$$

the entire experimental range (0.1-1.0 m) falls well within the Fresnel region, justifying the use of scalar diffraction for propagation analysis. The lateral resolution can be approximated as:

$$\Delta x \approx \frac{\lambda z}{D} \qquad (S6)$$

Then, Δx approximates 5, 15 and 36 mm when z is set to 0.1, 0.3 and 0.7 m. Fig. S10a shows the Pearson correlation coefficient (PCC) between the reconstructed and target images as a function of distance. It could be observed that the PCC remains above 0.9 for distances between 0.2 and 0.5 m, reaching its peak at 0.3 m.

Three reconstructed images at different distances are shown in Figs. S10b-d. Specifically, at z = 0.3 m, the holographic image in Fig. S10b exhibits high contrast and consistent with the theoretical resolution, with the letter “T” clearly resolved. At z = 0.1 m, the reconstructed image in Fig. S10c appears fragmented and discontinuous, despite a smaller theoretical Δx. This is due to strong Fresnel phase variations at the aperture, leading to incomplete constructive interference. At z = 0.7 m, the image Fig. S10d becomes blurred and peripheral ring-like artifacts emerge. This is attributed to reduced resolution, enhanced lateral spreading of diffracted waves under the finite aperture, and accumulated model deviations from scalar diffraction-based optimization.

These results indicate that an imaging distance of 0.3 m is effective for demonstrating microwave holographic imaging with our metasurface. At this distance, severe near-field distortions are avoided, while sufficient resolution and image fidelity are maintained, in agreement with both theoretical derivation and numerical simulations.

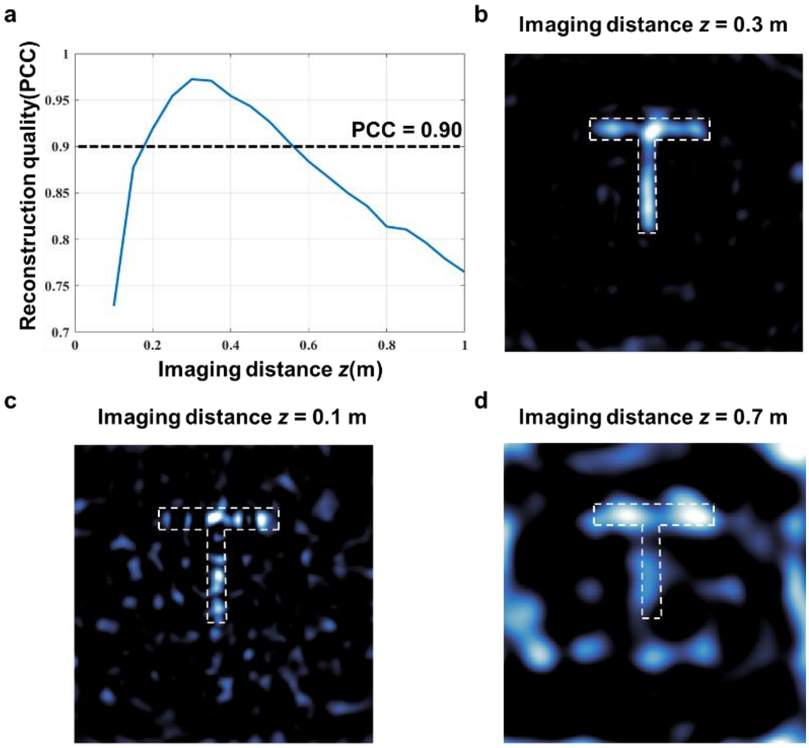

Fig. S10 | Impact of imaging distance on holographic image quality. a Pearson correlation coefficient (PCC) between the reconstructed and target images as a function of imaging distance. **b** Reconstructed letter “T” at z=0.3 m. **c** Reconstructed letter “T” at z=0.1 m. **d** Reconstructed letter “T” at z=0.7 m.

Supplementary Note 12. Performance of vital sign sensing under different practical conditions

We conducted additional experiments under various practical conditions to further evaluate the robustness of the metasurface-based human vital sign sensing approach.

(a) Subject distance and focal spot size:

The human respiration sensing results when the subject stood at 0.3 m in front of the metasurface have been presented in the main text. To further investigate the impact of subject distance, additional experiments were performed with the subject located at 0.1 m and 0.5 m, respectively. Figs. S11a-b show the measured electric field distributions and the extracted vital sign signals under the two distances. The experimental results indicated that the proposed metasurface-based approach consistently maintained high sensing performance as the target depth increased, enabling reliable estimation of human respiration rates. Additionally, the full width at half maximum (FWHM) was employed to quantify the focal spot size. Specifically, the focal spot measured 24.8 mm at 0.1 m and expanded to 68.6 mm at 0.5 m. As expected, the focal spot enlarged with distance due to diffraction and scattering during propagation. Nevertheless, the extracted respiration and heartbeat signals remained highly consistent with the benchmark values, confirming that the system maintained stable performance across different distances.

(b) Clothing effects:

To evaluate the influence of clothing, experiments were conducted with the subject wearing different types of clothes, including light clothing (T-shirt) and a thicker cotton coat. As shown in Fig. S11c, the subject stood 0.3 m in front of the metasurface while respiratory signals were recorded under each clothing condition. Although fabric absorption and scattering caused minor variations in the received signal amplitude, the reconstructed vital sign waveforms remained clear and accurate. These results demonstrated that the proposed system could maintains strong robustness across varying clothing conditions.

(c) Mutual interference in multi-person scenarios:

We further investigated the impact of surrounding bystanders on the vital sign sensing performance of the target individual in multi-person scenarios, as shown in Fig. S11d. In this scenario, the target subject stood 0.3 m in front of the metasurface while a passerby moved nearby. Since the focal spot size at 0.3 m is approximately 50.4 mm, smaller than the width of the human thoracic cavity, the majority of the electromagnetic energy is concentrated on the target's chest. Consequently, the passerby's movement has minimal impact on the quality of the target's echo signal. As shown in Fig. S11d, the system successfully extracted the target subject's respiration signal even in the

presence of a walking bystander. In a word, the proposed metasurface-based vital sign sensing approach, leveraging its strong electromagnetic focusing capability, effectively mitigates interference among multiple targets.

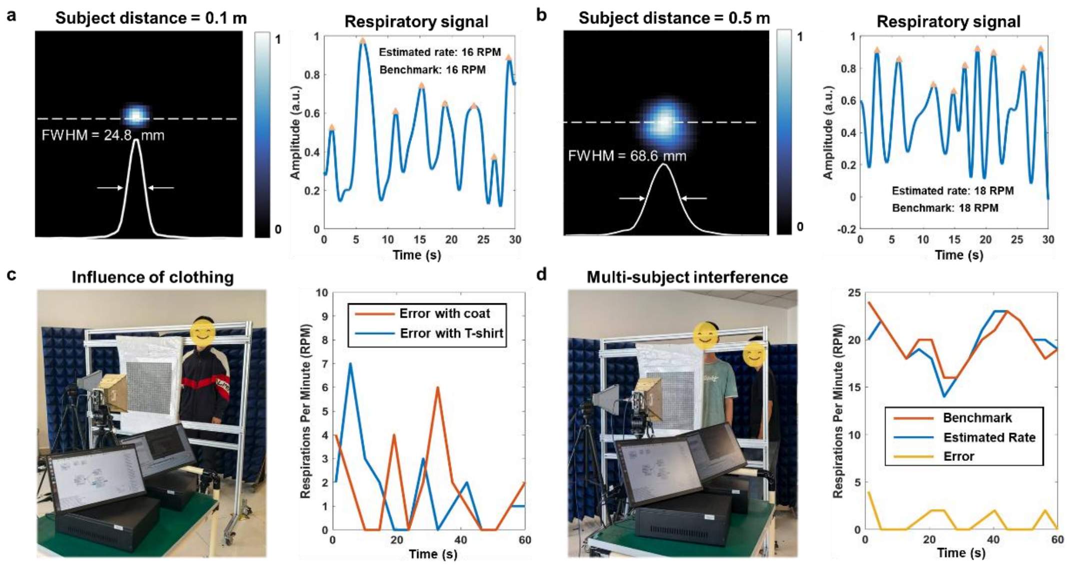

Fig. S11 | Vital sign sensing under different practical conditions. a Electric filed distribution and focal spot size at 0.1 m. **b** Electric filed distribution and focal spot size at 0.5 m. **c** Experimental setup and vital sign sensing result under different clothing conditions. **d** Experimental setup and vital sign sensing in the presence of multiple subjects.

Supplementary References

[S1] Lin, X. et al. All-optical machine learning using diffractive deep neural networks. *Science* **361**, 1004-1008 (2018).

[S2] Gu, Z. et al. Classification of Metal Handwritten Digits Based on Microwave Diffractive Deep Neural Network. *Advanced Optical Materials* **12**, 2301938 (2023).

[S3] Guo, Z. et al. Polarization-selective unidirectional and bidirectional diffractive neural networks for information security and sharing. *Nature Communications* **16**, 4492 (2025).
